# HSV-1 US3 Hijacks Conserved Actin Regulatory Complexes to Drive F-Actin Remodeling

**DOI:** 10.3390/v18070793

**Published:** 2026-07-19

**Authors:** Md Imran Hossain, Md Arifuzzaman, Md Mehedi Hasan, Seung-Jong Park, Leila Rahimian, Ojasvi Dutta, Vladimir Chouljenko, Harikrishnan Mohan, Reza Ghavimi, Konstantin G. Kousoulas

**Affiliations:** 1 Department of Pathobiological Sciences, Louisiana State University School of Veterinary Medicine, Baton Rouge, LA 70803, USA; mhoss52@lsu.edu (M.I.H.); mhasan10@lsu.edu (M.M.H.); lrahim1@lsu.edu (L.R.); odutta@lsu.edu (O.D.); vchoul1@lsu.edu (V.C.); hmohan1@lsu.edu (H.M.); rghavimi@lsu.edu (R.G.); 2 Division of Biotechnology and Molecular Medicine, School of Veterinary Medicine, Baton Rouge, LA 70803, USA; 3 Department of Computer Science, Missouri University of Science and Technology, Rolla, MO 65409, USA; marifuzzaman@mst.edu (M.A.); seung-jong.park@mst.edu (S.-J.P.)

**Keywords:** herpes simplex virus, US3 kinase, F-actin remodeling, protein complexes, machine learning, CORUM, cytoskeleton regulation

## Abstract

The herpes simplex virus 1 (HSV-1) US3 is a multifunctional serine/threonine kinase that promotes HSV-1 replication and spread. But its role and the mechanisms by which US3 regulates actin cytoskeletal remodeling remain poorly defined. We combined flow cytometry, confocal microscopy, immunoprecipitation-mass spectrometry (IP-MS), protein complex mapping, and machine learning to characterize US3-mediated F-actin dynamics. Flow cytometry and confocal microscopy showed that wild-type HSV-1 induces significant F-actin remodeling, while the ΔUS3 mutant displays F-actin levels comparable to uninfected cells, identifying US3 as a key regulator. IP-MS identified 47 high-confidence US3 interactors enriched in conserved actin regulatory complexes, including Arp2/3 nucleation machinery, formin-associated assemblies, cofilin severing complexes, and Rho-family GTPase modules. Mapping interactors to the CORUM database revealed clustering within actin nucleation, polymerization, and severing complexes, indicating that US3 operates through organized cellular machines. Machine-learning classifiers trained on label-independent mass-spectrometry features were used to prioritize interactors resembling known actin regulators; under protein-group-aware cross-validation, logistic regression performed best (average precision 0.24; ROC-AUC 0.66), and the analysis was interpreted as prioritization rather than de novo discovery. Pharmacological inhibition of Arp2/3 and formin pathways significantly attenuated US3-dependent F-actin remodeling, supporting the functional involvement of these pathways. Together, these findings are consistent with an inferred hierarchical axis in which US3 modulates Rho GTPase signaling and cofilin activation to promote F-actin disassembly, coordinating cytoskeletal remodeling required for efficient viral egress and spread.

## 1. Introduction

Herpes simplex virus type 1 (HSV-1) is a large enveloped DNA virus that requires dynamic interactions with host cell machinery for entry, intracellular transport, virion assembly, and egress [1]. The actin cytoskeleton, composed of dynamically polymerized and depolymerized actin filaments (F-actin), serves as a critical molecular scaffold for these processes, facilitating viral particle trafficking to replication compartments and supporting the membrane dynamics required for virion assembly and release [2,3,4]. Productive HSV-1 infection therefore demands extensive manipulation of host cellular processes, including the actin network [5,6]. HSV-1 entry and egress are both facilitated by actin-based structures; for example, infection induces filopodia (actin-rich membrane protrusions) that enhance virion attachment and promote spread to neighboring cells [7,8]. US3-driven actin remodeling occurs alongside its suppression of host innate immune responses, suggesting that these processes may collectively contribute to HSV-1 pathogenesis [9,10,11].

HSV-1 encodes a serine/threonine protein kinase, US3, that mediates cytoskeletal alterations during infection [9,12]. This protein is conserved among all members of the alphaherpesvirus subfamily and performs multiple functions, including inhibition of apoptosis, promotion of nuclear egress of virions, and modulation of host cell signaling [9,13,14]. It induces dramatic rearrangements of the actin cytoskeleton that correlate directly with enhanced viral spread. In related alphaherpesviruses such as pseudorabies virus (PRV), the US3 kinase has been shown to activate the host actin-severing protein cofilin by altering its phosphorylation state, leading to the breakdown of actin stress fibers and the formation of elongated cell membrane projections that facilitate direct cell-to-cell transmission [15,16]. US3 engages multiple host signaling cascades to achieve this effect; it can bind and phosphorylate p21-activated kinases (PAKs) and, through the canonical PAK–LIMK–cofilin pathway, modulate actin filament dynamics to promote efficient viral spread [15,17,18]. Beyond the cofilin pathway, US3 has been implicated in regulating additional cytoskeletal components; for example, the US3 protein of HSV-2 directly phosphorylates cytokeratin-17, contributing to F-actin disassembly and altered cell morphology [17,19]. Collectively, prior studies establish US3 as a multifunctional viral kinase that commandeers host actin remodeling machinery to enhance viral replication and dissemination.

Despite these insights, a comprehensive, systems-level understanding of how HSV-1 US3 interfaces with the host proteome to coordinate cytoskeletal remodeling remains incomplete. Most prior work has focused on individual pathways, such as the cofilin cascade, or relied on surrogate systems including overexpression or related alphaherpesviruses, and the full repertoire of host proteins physically associated with HSV-1 US3 during authentic infection remains largely undefined. It is also unclear whether US3′s manipulation of F-actin is restricted to known targets such as cofilin and PAKs or extends to a broader network of actin regulators, and whether US3-targeted proteins act as isolated factors or as components of defined mammalian protein complexes—multi-subunit machines that collectively govern actin dynamics. Resolving these higher-order organizational principles is essential for understanding how a single viral kinase achieves coordinated cytoskeletal remodeling and may reveal evolutionary constraints on viral substrate selection.

To address these questions, we used an integrative approach combining quantitative microscopy, interactomics, machine learning-based prediction, and pharmacological perturbation of actin regulatory pathways. We first used flow cytometry and confocal microscopy to quantify and visualize the effect of US3 on F-actin levels and three-dimensional organization in infected cells. We then identified host proteins that physically associate with US3 by immunoprecipitation followed by mass spectrometry (IP-MS) and mapped these interactors onto the Comprehensive Resource of Mammalian Protein Complexes (CORUM) to determine whether US3-targeted proteins cluster within known functional complexes. Network analysis was used to visualize connectivity and functional enrichment among US3-associated proteins, with emphasis on actin regulatory modules. We then applied three machine-learning models (logistic regression, random forest, and gradient-boosted trees), trained on label-independent mass-spectrometry features, to prioritize interactors most likely to resemble known actin regulators. Finally, we used Arp2/3 and formin small-molecule inhibitors to test whether the predicted regulatory complexes contribute to US3-dependent F-actin remodeling. By integrating quantitative microscopy, global interactomics, protein complex annotation, machine learning, and pharmacological validation, we provide a systems-level view of how HSV-1 US3 engages conserved mammalian protein complexes to remodel F-actin, and identify candidate cellular targets for limiting HSV-1 transport and spread.

## 2. Methodology

### 2.1. Cell and Cell Culture

African green monkey kidney (Vero; ATCC CCL-81) and Neuro-2A (N2A; ATCC CCL-131) cells were obtained from the American Type Culture Collection (ATCC, Manassas, VA, USA). Cells werecultured in Dulbecco’s Modified Eagle Medium (DMEM; Gibco, Thermo Fisher Scientific, Waltham, MA, USA) supplemented with 10% fetal bovine serum (FBS) and 1% penicillin/streptomycin (Gibco, Thermo Fisher Scientific, Waltham, MA, USA) at 37 °C with 5% CO_2_. Cells were passaged every 3–4 days at 80–90% confluence using 0.25% trypsin–EDTA (Gibco, Thermo Fisher Scientific, Waltham, MA, USA).

### 2.2. Viruses

A pathogenic HSV-1 (McKrae) bacterial artificial chromosome (BAC) strain expressing green fluorescent protein (GFP) was used as the wild-type virus. An isogenic HSV-1 (McKrae) ΔUS3 mutant lacking the US3 open reading frame was generated in our laboratory using markerless two-step Red-mediated recombination as previously described [20,21]. Briefly, the infectious HSV-1 (McKrae) BAC was propagated in Escherichia coli strain SW105, which encodes arabinose-inducible λ Red recombination functions and FLP recombinase. For US3 deletion, PCR-amplified DNA fragments containing upstream and downstream homology arms flanking the US3 locus were used to direct homologous recombination. A kanamycin resistance cassette flanked by FLP recombination target (FRT) sites was first inserted in place of the US3 coding region following induction of λ Red recombination with L-arabinose. Recombinant clones were selected on kanamycin-containing agar plates and screened by colony PCR to verify correct targeting of the US3 locus. In the second recombination step, FLP recombinase was induced to excise the kanamycin cassette, resulting in a markerless deletion of the US3 open reading frame without disrupting adjacent viral sequences. Precise deletion and cassette excision were confirmed by PCR and Sanger sequencing across the modified genomic region. Oligonucleotides used for mutant construction are provided in Supplementary Table S1.

Purified BAC DNA containing the ΔUS3 genome was transfected into Vero cells using Lipofectamine 3000 (Invitrogen, Thermo Fisher Scientific, Waltham, MA, USA) to reconstitute infectious virus. Viral stocks were subsequently amplified in Vero cells, and loss of US3 protein expression in ΔUS3-infected cells was verified by immunoblotting. Viral titers were determined by standard plaque assays on Vero cell monolayers. Oligonucleotides used for BAC engineering and related constructs are listed in Supplementary Table S1.

### 2.3. Flow Cytometry for F-Actin Quantification and Pharmacological Inhibition Assays

To evaluate whether HSV-1 US3 modulates actin cytoskeletal dynamics during infection, N2A cells were infected with wild-type HSV-1 (McKrae) BAC, an isogenic ΔUS3 HSV-1 mutant (generated via two-step λ Red recombination in the McKrae BAC backbone), or mock-treated. N2A cells were seeded in 6-well plates at 2 × 10^5^ cells per well and infected at a multiplicity of infection (MOI) of 10. To pharmacologically validate the actin regulatory pathways identified through machine-learning analysis, cells were treated with inhibitors targeting major actin polymerization systems. Specifically, the formin inhibitor SMIFH2 (MedChemExpress, Monmouth Junction, NJ, USA; 20 μM) and the Arp2/3 complex inhibitor CK-666 (MedChemExpress, Monmouth Junction, NJ, USA;100 μM) were used. These concentrations were selected based on previous studies demonstrating effective inhibition of formin and Arp2/3-dependent actin polymerization without significant cytotoxicity [22,23,24]. Cells were pretreated with inhibitors for 30 min prior to viral infection, and the inhibitors were maintained in the culture medium throughout the infection period. Control cells received an equivalent volume of dimethyl sulfoxide (DMSO; Fisher Scientific, Hampton, NH, USA) as the vehicle control.At the indicated time points post-infection, cells were detached using trypsin, washed with PBS, stained with Viobility™ 405/520 Fixable Dye (Miltenyi Biotec; Cat: 130-109-814, Bergisch Gladbach, Germany), and fixed in 4% paraformaldehyde for 10 min at room temperature. Cells were then permeabilized with 0.1% Triton X-100 for 10 min, followed by staining with Alexa Fluor 647–conjugated phalloidin (Thermo Fisher Scientific, Waltham, MA, USA; Cat. No. A22287; 1:200 dilution) to quantify filamentous actin (F-actin) for 30 min at room temperature in PBS containing 1% bovine serum albumin (BSA).

Samples were analyzed on a BD FACSCanto II flow cytometer using FACSDiva (BD Biosciences, San Jose, CA, USA). Data were processed using FlowJo v10.8.1 with the following gating strategy: FSC-A vs. SSC-A to select the main cell population, FSC-A vs. FSC-H to exclude doublets, viability dye to gate live cells, and Alexa Fluor 647 signal to quantify F-actin levels. F-actin abundance was quantified as the geometric mean fluorescence intensity (GeoMean MFI) of Alexa Fluor 647 within the live singlet population. Experiments were performed with three independent biological replicates per condition. Statistical analysis was conducted using two-way analysis of variance (ANOVA) followed by Šidák’s multiple comparisons test in GraphPad Prism v9.5.1. Data are presented as mean ± SEM, and differences were considered statistically significant when *p* < 0.05. Antibodies and reagents are listed in the Supplementary Table S2.

### 2.4. Confocal Microscopy and Quantitative Confocal Image Analysis

N2A cells were seeded onto µ-Slide 8-well glass-bottom chambers (ibidi GmbH, Gräfelfing, Germany; Cat. No. 80806)and infected as described above. At 0 or 24 h post-infection (hpi), cells were fixed in 4% paraformaldehyde (PFA) for 15 min at room temperature and washed with phosphate-buffered saline (PBS). Cells were permeabilized with 0.1% Triton X-100 in PBS for 10 min and blocked in PBS containing 2% bovine serum albumin (BSA) for 1 h at room temperature to reduce nonspecific staining.

F-actin was visualized using Alexa Fluor™ 488–conjugated phalloidin (Invitrogen, Thermo Fisher Scientific, Waltham, MA, USA; 1:200 dilution) for 30 min at room temperature in the dark. After washing, samples were mounted using Duolink^®^ In Situ Mounting Medium with DAPI (Sigma-Aldrich, St. Louis, MO, USA; Cat. No. DUO82040) to counterstain nuclei. Images were acquired using an Olympus FLUOVIEW™ FV3000 confocal laser scanning microscope (Olympus Corporation, Tokyo, Japan) equipped with a 100× oil-immersion objective. Identical laser power, detector gain, and acquisition settings were maintained across all experimental conditions. Representative images are shown from three independent biological replicates. Antibodies and reagents are listed in the Supplementary Table S2.

Raw, unprocessed confocal images were analyzed using Fiji/ImageJ (version 2.16.0/1.54p). Images were acquired using identical laser power, detector gain, pinhole, objective, pixel dimensions, and acquisition settings across all experimental conditions. The Alexa Fluor 488–phalloidin channel was separated from the DAPI channel and analyzed as a grayscale image. Complete, non-overlapping cells that were not truncated by the image boundary were manually outlined using the freehand selection tool and saved as regions of interest (ROIs). Image files were coded before analysis to minimize selection bias.

For each image, background fluorescence was determined from three cell-free regions. Background-corrected mean F-actin fluorescence intensity was calculated for each cell as

Corrected mean fluorescence=Mean cellular fluorescence−Mean background fluorescence

For quantification of F-actin-positive area, a duplicate of the phalloidin channel was used. The same background-subtraction and thresholding parameters were applied to all images. A fixed intensity threshold was selected before decoding the experimental groups and was maintained across all conditions. Within each cell ROI, the F-actin-positive area fraction was calculated as

$$F\text{-}\mathrm{actin}\;\mathrm{area}\;\mathrm{fraction}\;(\%)=\frac{\mathrm{Threshold}\text{-}\mathrm{positive}\;\mathrm{area}}{\mathrm{Total}\;\mathrm{cellular}\;\mathrm{ROI}\;\mathrm{area}}\times 100$$

The nuclear region was excluded from the area-fraction analysis using the corresponding DAPI image. Mean fluorescence intensity and F-actin area-fraction values were log_10_-transformed before graphical presentation and statistical analysis. Multiple cells from several randomly selected fields were analyzed for each condition in each independent experiment. Cell-level measurements were averaged within each biological replicate, and biological-replicate means were used for statistical comparisons.

### 2.5. Immunoprecipitation and Mass Spectrometry Analysis

For immunoprecipitation experiments, Vero cells were infected with HSV-1 (McKrae) at a multiplicity of infection (MOI) of 10. At 24 h post-infection (hpi), cells were harvested and lysed in NP-40 lysis buffer (50 mM Tris-HCl, pH 7.4; 150 mM NaCl; 1% NP-40; 5 mM EDTA) supplemented with protease and phosphatase inhibitor cocktails. Lysates were clarified by centrifugation at 13,000 rpm for 10 min at 4 °C, and supernatants were collected for downstream immunoprecipitation.

Immunoprecipitation was performed using Protein G magnetic Dynabeads™ (Invitrogen, Thermo Fisher Scientific, Waltham, MA, USA; Cat. No. 10007D) according to the manufacturer’s instructions. Dynabeads were incubated overnight at 4 °C with rabbit anti-US3 antibody (Abcam, Cambridge, UK; Cat. No. ab92392) to allow antibody coupling. Antibody-bound beads were washed three times and incubated overnight at 4 °C with clarified cell lysates under gentle rotation. Beads were subsequently washed three times to remove nonspecifically bound proteins. Immune complexes, together with the beads, were submitted to the IDeA National Resource for Quantitative Proteomics (University of Arkansas for Medical Sciences, Little Rock, AR, USA) for downstream proteomic processing and LC–MS/MS analysis. All primary antibodies and key reagents are summarized in Supplementary Table S2.

Peptides were analyzed by liquid chromatography–tandem mass spectrometry (LC–MS/MS). Raw mass spectrometry data were processed using Scaffold DDA™ (version 6.7.2; Proteome Software Inc., Portland, OR, USA) for peptide and protein identification. Database searches were conducted against the human UniProt/SwissProt reference proteome supplemented with the HSV-1 (McKrae) proteome. Peptide-spectrum matches (PSMs) and protein identifications were filtered to achieve a false discovery rate (FDR) of <1% at both the peptide and protein levels, with a minimum requirement of two unique peptides per protein.

Label-free spectral quantification was used to compare US3 immunoprecipitation samples to mock (IgG control) immunoprecipitations. High-confidence interactors were defined as proteins enriched in US3 pull-down samples with log_2_(US3/mock) > 2 and adjusted *p* < 0.01 (moderated *t*-test; *n* = 3 biological replicates). Common contaminants, reverse hits, and decoy sequences were excluded from analysis.

### 2.6. Functional Enrichment and Network Analysis with Co-Immunoprecipitation and Reciprocal Pull-Down Validation

Gene Ontology (GO) enrichment analysis was performed using ClueGO v2.5.9 within Cytoscape v3.10.2 [25,26,27,28]. Enrichment significance was calculated using a right-sided hypergeometric test with Benjamini–Hochberg false discovery rate (FDR) correction (q < 0.05). A kappa similarity threshold of 0.3 was applied to functional group GO terms. Complementary enrichment analysis was conducted using g:Profiler (g:GOSt mode; organism: *Homo sapiens*), considering GO biological process (BP), molecular function (MF), cellular component (CC), KEGG pathways, and Human Phenotype (HP) terms (FDR < 0.05) [29,30,31]. Manhattan plots and summary bar charts were generated to visualize enrichment results. Protein complex mapping was performed using the CORUM v4.0 database. Complex coverage was calculated as follows: (number of US3 interactors within a complex)/(total annotated subunits of that complex). Interaction networks were visualized in Cytoscape using the organic layout algorithm. Node size and color were scaled according to log_10_(FDR-adjusted *p*-value), and functionally related terms were grouped based on kappa similarity.

Co-Immunoprecipitation and Reciprocal Pull-Down Validation:

Physical interactions between US3 and candidate partners were validated by reciprocal co-immunoprecipitation. Vero cells were infected with HSV-1 strain McKrae at a multiplicity of infection (MOI) of 10. At 24 h post-infection, cells were harvested and lysed in NP-40 lysis buffer (50 mM Tris-HCl, pH 7.4; 150 mM NaCl; 1% NP-40; 5 mM EDTA) supplemented with protease and phosphatase inhibitor tablets. Lysates were clarified by centrifugation at 13,000 rpm for 10 min at 4 °C and the supernatants collected.

Immunoprecipitation was performed using Protein G magnetic Dynabeads (Invitrogen, Thermo Fisher Scientific, Waltham, MA, USA; Cat. No. 10007D)according to the manufacturer’s instructions. Dynabeads were incubated overnight at 4 °C with the appropriate primary antibody to allow antibody coupling. For reciprocal pull-downs, the bait antibody was rabbit anti-US3 (Abcam, Cambridge, UK; Cat. No. ab92392)in one direction and rabbit anti-β-catenin (Cell Signaling Technology, Danvers, MA, USA; Cat. No. 9562) in the reciprocal direction, with a rabbit polyclonal isotype control (Abcam, ab171870) run in parallel; mock-lysate immunoprecipitations served as an additional specificity control. Antibody-bound beads were washed three times with antibody binding and washing buffer, then incubated overnight at 4 °C with clarified cell lysates under gentle rotation. Beads were washed three times to remove unbound proteins, and bound immune complexes were eluted in 40 μL of elution buffer and analyzed by Western blotting. All primary antibodies and key reagents are summarized in Supplementary Table S2.

### 2.7. Machine Learning for F-Actin Regulator Prioritization: Dataset and Feature Construction

A structured protein-level dataset of 639 proteins was assembled from the complete mass-spectrometry dataset to support supervised prioritization of candidate F-actin regulators. This dataset included the 47 proteins that met the predefined high-confidence US3-interactor criteria, as well as additional proteins detected or retained in the broader mass-spectrometry dataset. The 639-protein dataset was used for model training and evaluation, whereas the 47 high-confidence interactors represented the primary experimentally supported candidates for biological interpretation. Proteins prioritized computationally but not meeting the high-confidence IP-MS criteria were treated as predicted or reference candidates and were not considered experimentally validated US3 interactors. Curated annotations Gene Ontology actin association (GO Actin), functional annotations for actin polymerization, depolymerization, and dual regulation, and evidence categories (literature-supported, experimentally validated, computationally predicted, and indirect actin regulation) were compiled from UniProt, PubMed, Reactome, and GO. These curated annotations were used solely to define the class label (direct F-actin regulator versus non-regulator): proteins with experimentally supported direct modulation of filamentous actin polymerization or depolymerization were labeled regulators, and all others non-regulators.

Model features were derived entirely from the mass-spectrometry experiment and were independent of these annotation labels. For each protein, six label-independent features were computed from the US3 immunoprecipitation dataset: pull-down exclusivity, log_2_ enrichment of the US3 immunoprecipitation over the control immunoprecipitation for spectral counts and for precursor intensity, US3 sample spectral count, US3 sample unique peptide count, and absence in the control immunoprecipitation. Annotation-derived variables were explicitly excluded from the feature set to prevent circularity between features and labels. The feature matrix and class labels are provided in Supplementary Data S4.

### 2.8. Model Training and Validation

Supervised classification was performed using stratified 5-fold cross-validation to preserve class balance across training and test splits. In each fold, 80% of the proteins were used for model training, and 20% were held out for independent testing. Because several proteins shared identical mass-spectrometry feature profiles (shared peptide protein groups), supervised evaluation used protein-group-aware stratified cross-validation (StratifiedGroupKFold, 5 folds), keeping identical-profile proteins within the same fold to prevent information leakage. Feature scaling was fitted within training folds only.

All feature preprocessing steps were performed within training folds to prevent data leakage. Three classifiers were evaluated: logistic regression (scikit-learn v1.5.2; L2 regularization, C = 1.0; class-weight balancing), random forest (600 trees; max_features = √p; class-weight balancing), and gradient-boosted trees (balanced sample weights). Features comprised six label-independent mass-spectrometry observables (pull-down exclusivity, log_2_ US3/control enrichment for spectral counts and precursor intensity, US3 spectral count, US3 unique peptides, and control-absence); annotation-derived variables were excluded from the feature set.

### 2.9. Performance Evaluation

Model performance was evaluated on held-out test sets using accuracy, precision, recall, Macro-F1 score, Matthews correlation coefficient (MCC), and area under the receiver operating characteristic curve (AUC-ROC) [32,33,34]. Performance metrics are reported as mean ± standard deviation across folds. Given the ~5% positive-class prevalence, average precision (AP) was the primary metric, with ROC-AUC secondary and MCC, F1, and balanced accuracy reported at a fixed 0.5 threshold. Models were compared using the Friedman test with Wilcoxon signed-rank post hoc across fold–seed values. Representative confusion matrices and ROC/precision–recall curves are shown in Section 3.

### 2.10. Statistical Analysis

All statistical analyses were performed using GraphPad Prism (v9.5.1; GraphPad Software) or Python (scipy.stats v1.11.1). Normality of data distributions was assessed using the Shapiro–Wilk test. Parametric tests were applied only when data satisfied normality assumptions; otherwise, appropriate nonparametric tests were used as indicated in the corresponding figure legends. For comparisons between two groups, unpaired two-tailed *t*-tests were used when assumptions were met. For multi-group or time-course experiments, two-way analysis of variance (ANOVA) followed by Šidák’s multiple-comparisons test was performed as specified. Machine-learning models were compared using the Friedman test followed by Wilcoxon signed-rank tests for pairwise comparisons. Unless otherwise indicated, data are presented as mean ± standard error of the mean (SEM). The term n refers to independent biological replicates. No statistical methods were used to predetermine sample size. No data points were excluded from analysis. Investigators were not blinded to experimental group allocation during data collection and analysis. A two-sided *p* value < 0.05 was considered statistically significant. For proteomic and enrichment analyses, false discovery rate (FDR)-adjusted q values < 0.05 were considered significant.

## 3. Results

### 3.1. HSV-1 US3 Drives Biphasic F-Actin Remodeling During Infection

**Figure 1:**
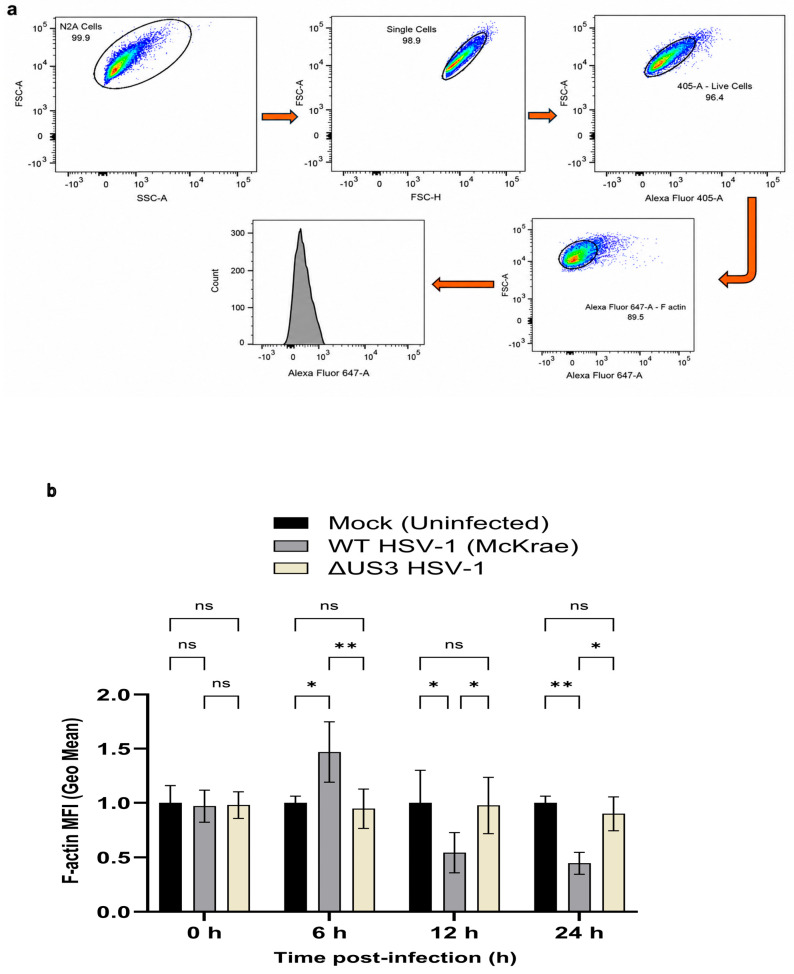
US3 is required for dynamic F-actin remodeling during HSV-1 infection. (**a**) Flow-cytometry gating strategy. N2A cells were gated on FSC-A vs. SSC-A (cells), FSC-A vs. FSC-H (singlets), viability dye (live cells), and Alexa Fluor 647-phalloidin (F-actin GeoMean). (**b**) Quantification of F-actin GeoMean across time (0, 6, 12, 24 hpi) from three independent experiments. Note that 0 hpi refers to the time immediately after the attachment step. Data are mean ± SEM. Two-way ANOVA with Tukey multiple comparisons test; * *p* < 0.05, ** *p* < 0.01, ns = not significant.

To determine whether US3 modulates actin cytoskeletal dynamics during infection, Neuro-2A (N2A) cells were infected with WT HSV-1 (McKrae) BAC, an isogenic deltaUS3 mutant virus generated by two-step lambda Red recombination in the McKrae BAC backbone, or mock-treated. F-actin was quantified by flow cytometry using Alexa Fluor 647-phalloidin. Cells were sequentially gated on FSC-A versus SSC-A to select the main population, FSC-A versus FSC-H to exclude doublets, and a 405 nm fixable viability dye to gate live cells; F-actin abundance was quantified within the live singlet gate as the GeoMean MFI of Alexa 647 (Figure 1a). The 0 hpi sample was harvested immediately after the 1 h adsorption step and captures any contribution from tegument-delivered US3 prior to de novo expression.

WT HSV-1 infection produced a striking biphasic F-actin response, with significant early accumulation at 6 hpi (+47% vs. mock; 774 vs. 527 GeoMean MFI; Tukey *p* = 0.016), followed by pronounced late-stage depletion at 12 hpi (−46%; 311 MFI; *p* = 0.011) and 24 hpi (−55%; 254 MFI; *p* = 0.0023) (Figure 1b). In contrast, deltaUS3-infected cells showed no measurable deviation from mock controls at any time point (all Tukey-adjusted *p* > 0.77), demonstrating that US3 is required for the temporal reprogramming of actin dynamics during infection. Two-way ANOVA revealed a highly significant time x virus interaction (F_{3,24} = 14.2, *p* < 0.0001), confirming that the observed cytoskeletal remodeling is US3-dependent rather than a consequence of generalized viral replication (*n* = 3 biological replicates per condition; full ANOVA output in Supplementary Data S1). Collectively, these data demonstrate that US3 orchestrates a temporal switch from early actin stabilization to late actin disassembly, consistent with stage-specific modulation of cytoskeletal architecture during HSV-1 infection.

**Figure 2:**
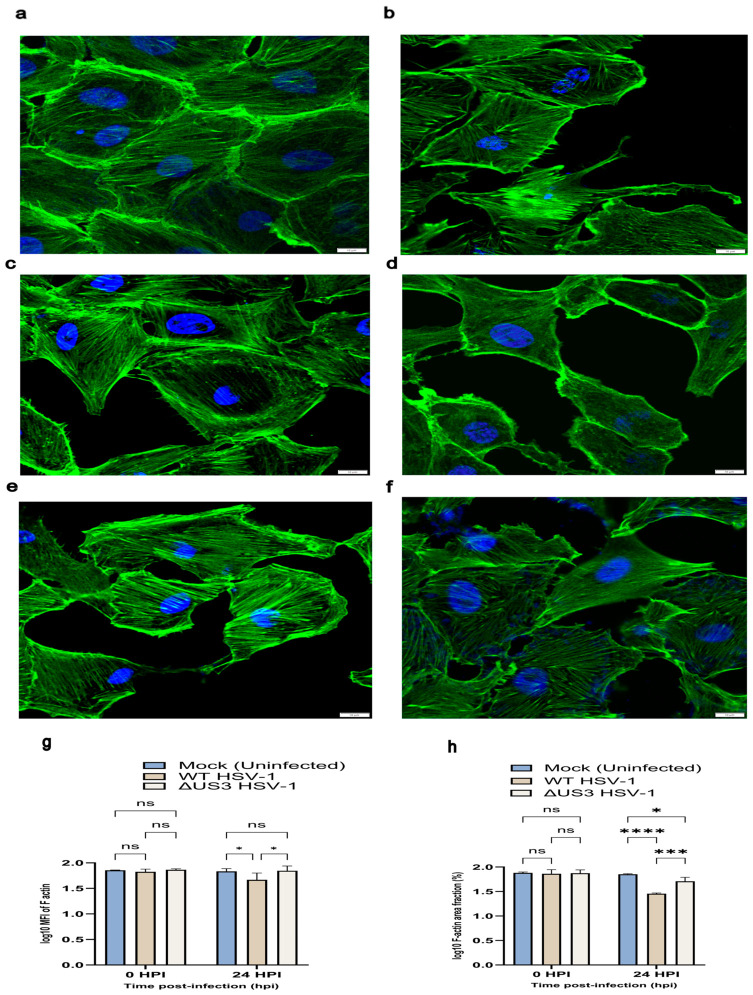
Confocal imaging shows US3-dependent loss of filamentous actin at late infection. N2A cells were infected with WT HSV-1, ΔUS3 HSV-1, or mock-infected, fixed at 0 or 24 hpi, and stained with Alexa Fluor 488–phalloidin (F-actin, green) and DAPI (nuclei, blue). Scale bars, 10 μm. (**a**) Mock-infected F-actin stress fiber morphology at 0 hpi. (**b**) Mock-infected F-actin stress fiber morphology at 24 hpi. (**c**) WT HSV-1-infected F-actin stress fiber morphology at 0 hpi. (**d**) WT HSV-1-infected F-actin stress fiber morphology at 24 hpi. (**e**) ΔUS3 HSV-1-infected F-actin stress fiber morphology at 0 hpi. (**f**) ΔUS3 HSV-1 infected F-actin stress fiber morphology at 24 hpi. (**g**) Quantification of total F-actin content (log_10_ mean fluorescence intensity, MFI) and (**h**) F-actin area fraction (log_10_%) from confocal images in (**a**–**f**), measured in Fiji/ImageJ. Cells were segmented and background-subtracted, and images were coded and analyzed blind to condition. Bars represent mean ± SEM of three independent biological replicates. Statistical significance was assessed by two-way ANOVA with Tukey’s multiple-comparisons test. ns, not significant; * *p* < 0.05; *** *p* < 0.001; **** *p* < 0.0001.

To visualize the impact of US3 on F-actin architecture, confocal microscopy was performed on N2A cells infected with WT HSV-1, deltaUS3, or mock at MOI = 10, fixed at 0 or 24 hpi, and stained with Alexa Fluor 488 phalloidin (F-actin, green) and DAPI (nuclei, blue). At 0 hpi (Figure 2a,c,e), all conditions displayed robust stress fibers spanning the cell body and dense cortical actin at the periphery. By 24 hpi, WT HSV-1-infected cells showed marked remodeling, with prominent stress fiber disassembly and retention of only diffuse cortical actin (Figure 2d). In contrast, mock and deltaUS3 cells retained relatively unchanged morphology with regular stress fiber patterns and minimal cortical elaboration (Figure 2b,f). These images support a model in which US3 promotes F-actin accumulation at cortical and perinuclear regions during early to mid-infection—facilitating transport and replication compartment dynamics—followed by late-stage actin disassembly that enables nuclear envelope deformation for egress and extension of actin-based projections for spread. The preservation of some stress fibers at 24 hpi in WT-infected cells indicates that US3 selectively modulates actin architecture rather than driving global depolymerization, consistent with fine-tuned regulation via actin regulatory complexes.

To complement the flow-cytometry data with spatial quantification, F-actin in confocal images was measured in Fiji as both total content (MFI) and area fraction across three biological replicates (Figure 2g,h). At 0 hpi, all conditions were statistically indistinguishable for both metrics (Tukey *p* > 0.7). By 24 hpi, WT HSV-1 significantly reduced F-actin by both measures—MFI (mean 1.668 vs. 1.833 for mock; *p* = 0.0434) and area fraction (1.456 vs. 1.853; *p* < 0.0001)—whereas ΔUS3 retained mock-level MFI (1.846; *p* = 0.97 vs. mock) and differed significantly from WT (*p* = 0.0295). Area fraction showed the same WT-dependent loss (WT vs. ΔUS3, *p* = 0.0004) but also a smaller reduction in ΔUS3 relative to mock (1.710 vs. 1.853; *p* = 0.0235), indicating that while the major F-actin depletion at 24 hpi is US3-dependent, a minor component of the spatial reduction occurs independently of US3. These quantitative results corroborate the flow-cytometry findings and confirm that US3 is the principal driver of late-stage F-actin loss.

### 3.2. US3 Associates with Host Actin Regulatory and Trafficking Proteins

To identify host factors mediating this effect, we performed IP-MS using Vero cells, which support robust HSV-1 replication and yield sufficient material for stringent IP workflows. Vero cells were infected with WT HSV-1 and subjected to anti-US3 immunoprecipitation at 24 hpi. Eluted proteins were analyzed by liquid chromatography–tandem mass spectrometry (LC-MS/MS). After filtering against parallel IgG isotype control immunoprecipitations (mock IP) and applying a stringent false discovery rate (FDR < 0.01), we identified 47 high-confidence US3-interacting host proteins.

**Figure 3:**
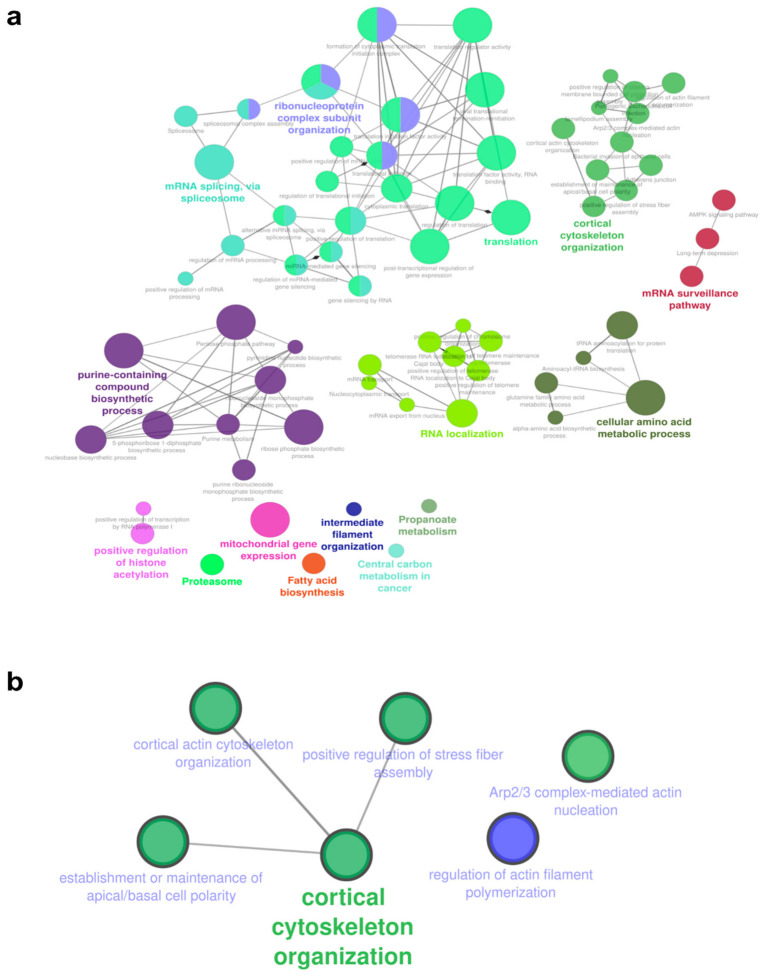
ClueGO network analysis reveals US3 interactors enriched in cortical actin-remodeling pathways. (**a**) Functional network of GO terms enriched among high-confidence US3-interacting host proteins (ClueGO analysis) from US3 precipitated mass spec analysis. Node size reflects enrichment significance; node color denotes functional modules. Cytoskeleton-related processes (green) form a dominant cluster centered on cortical cytoskeleton organization. (**b**) Actin-focused subnetwork highlighting terms directly linked to F-actin regulation, including cortical cytoskeleton organization, positive regulation of stress fiber assembly, Arp2/3 complex-mediated actin nucleation, and regulation of actin filament polymerization.

Functional enrichment analysis using ClueGO revealed that US3 interactors cluster into multiple biologically coherent functional modules, with a dominant network of cytoskeleton-related processes (Figure 3a, green nodes). Cortical cytoskeleton organization emerged as a central hub, interconnected with positive regulation of stress fiber assembly, Arp2/3 complex-mediated actin nucleation, and regulation of actin filament polymerization (Figure 3b). This clustering reflects mechanistic engagement of US3 with host pathways responsible for spatial control of actin remodeling at the cell periphery, a region critical for late-stage virion egress. Beyond cytoskeletal remodeling, additional clusters included RNA localization, translation, and ribonucleoprotein complex subunit organization, potentially reflecting US3′s broader role in modulating mRNA dynamics and translation during infection. However, these pathways are peripheral in the network compared with the actin-centric hubs.

Independent enrichment analysis with g:Profiler confirmed and refined this picture. In the global analysis of all US3-bound proteins, significantly enriched terms spanned molecular function, biological process, and cellular component categories, indicating that US3 targets proteins that are enzymatically active, spatially organized, and integrated within defined cytoskeletal compartments (Supplementary Figure S2a,c). Restricting the analysis to interactors annotated as actin regulators yielded highly specific enrichment for regulation of actin filament polymerization, Arp2/3 complex-mediated actin nucleation, cortical cytoskeleton organization, and stress fiber assembly (Supplementary Figure S2b,d). The tight functional coherence of these interactors indicates that US3 does not engage cytoskeletal proteins randomly but instead targets a defined network of actin regulatory modules that collectively account for the biphasic F-actin trajectory observed experimentally. Together, these analyses establish that HSV-1 US3 interacts with a spatially and functionally coherent network of proteins localized to the cortical actin cytoskeleton and responsible for actin filament nucleation, elongation, stabilization, and severing, providing a mechanistic basis for its role in promoting F-actin remodeling and facilitating viral egress and spread.

### 3.3. US3 Targets Multiprotein Assemblies Governing Actin Dynamics and Transport

**Figure 4:**
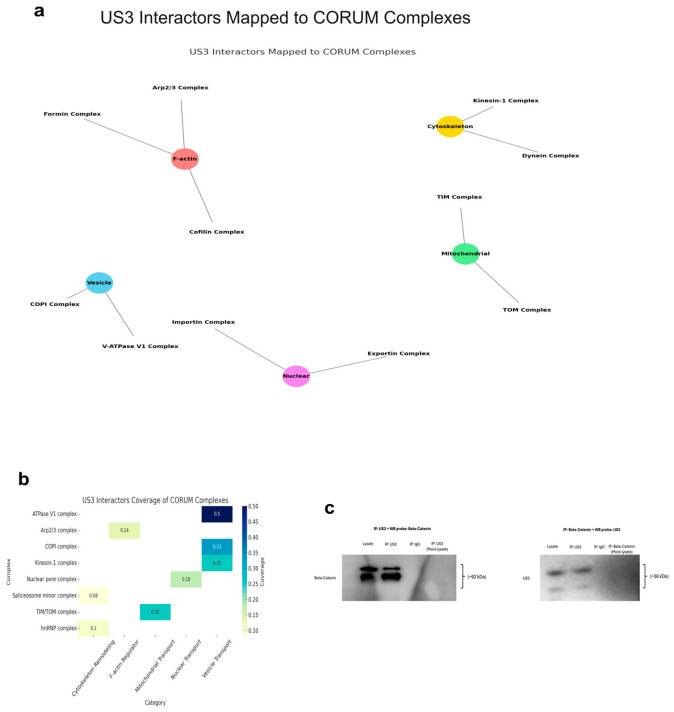
US3 interactors cluster within conserved CORUM protein complexes leading actin dynamics and transport. (**a**) Network representation of CORUM complexes containing at least one US3 interactor. Nodes represent protein complexes grouped by functional category (F-actin regulators, cytoskeleton, mitochondrial transport, nuclear transport, vesicle transport); edges connect complexes that share US3-bound subunits. (**b**) Heatmap of “coverage” values for each complex, defined as the fraction of its subunits detected as US3 interactors. High coverage of Arp2/3, kinesin-1, COPI, TIM/TOM, nuclear pore, and V-ATPase V1 complexes indicates that US3 interfaces with intact multiprotein machines rather than with isolated factors. (**c**) Reciprocal co-immunoprecipitation of US3 and β-catenin. β-Catenin was detected following US3 immunoprecipitation, and US3 was detected following β-catenin immunoprecipitation; IgG and mock-lysate controls showed no specific signal.

To determine whether US3-associated host proteins function as isolated factors or as components of higher-order molecular machines, we mapped the interactors onto the CORUM database of curated mammalian protein complexes. US3 interactors clustered within defined multi-subunit complexes rather than distributing randomly across unrelated pathways (Figure 4a). Multiple components of core actin regulatory assemblies were identified, including the Arp2/3 complex, formin-associated complexes, and cofilin-related severing complexes, all of which directly govern actin filament nucleation, elongation, and severing. In addition to cytoskeletal complexes, US3 interactors mapped to kinesin-1 and dynein motor complexes, COPI vesicle coat complexes, the V-ATPase V1 complex, nuclear pore-associated transport complexes (importin/exportin), and mitochondrial TIM/TOM complexes, indicating that US3 also engages intracellular trafficking and organelle-associated transport machinery. The spatial distribution of these complexes suggests that US3 simultaneously interfaces with actin remodeling modules and vesicular/motor systems that coordinate membrane dynamics and cargo movement.

To provide biochemical validation of the IP–MS results, the association between US3 and β-catenin was examined by reciprocal co-immunoprecipitation. β-Catenin was detected in US3 immunoprecipitates, and conversely, US3 was detected in β-catenin immunoprecipitates, whereas the IgG and mock-lysate controls showed no specific signal (Figure 4c). These findings support a specific association between US3 and β-catenin, although they do not by themselves establish a direct physical interaction.

Quantitative assessment of complex engagement was performed by calculating coverage values, defined as the fraction of subunits within each CORUM complex identified as US3 interactors (Figure 4b). Among cytoskeletal regulators, the Arp2/3 complex showed coverage of ~0.14, indicating that US3 associates with multiple components of this actin nucleation machinery. Kinesin-1 and TIM/TOM complexes exhibited coverage of ~0.25, while the COPI complex showed ~0.33. The V-ATPase V1 complex showed the highest coverage (~0.5), suggesting extensive engagement of the vesicular acidification and trafficking machinery. Although individual coverage values varied, the consistent presence of multiple subunits within the same assemblies indicates that US3 interfaces with intact multiprotein machines rather than with isolated factors.

### 3.4. Machine-Learning Classifiers Identify a US3-Driven F-Actin Remodeling Circuitry

**Figure 5:**
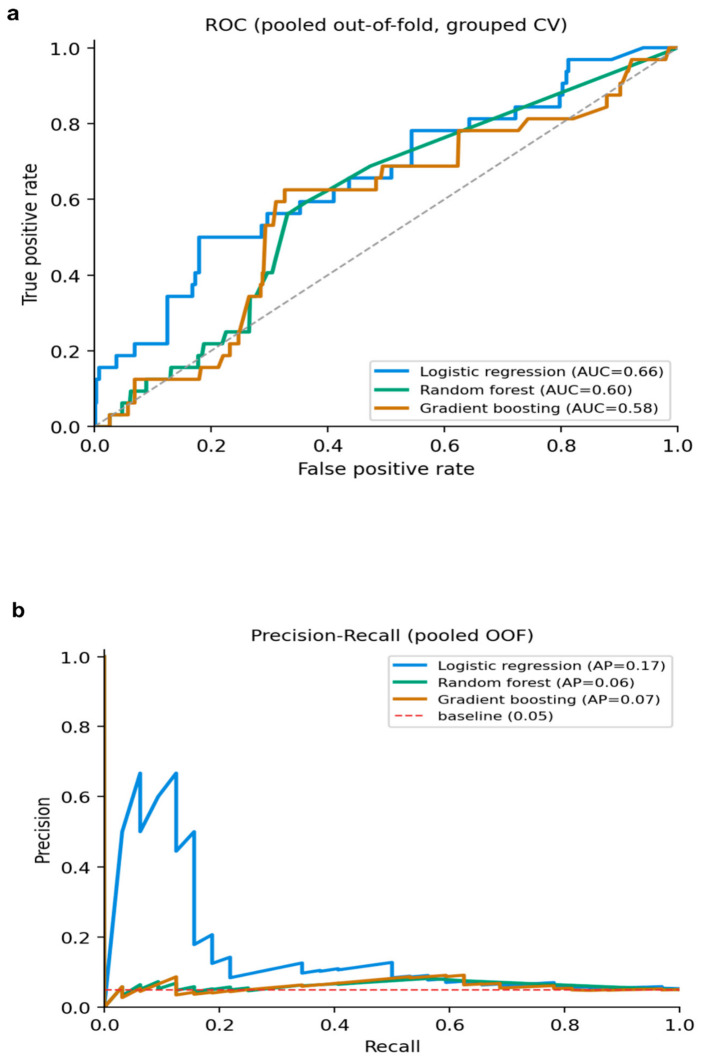
Machine-learning prioritization of US3 interactors using label-independent mass-spectrometry features. (**a**) Receiver-operating-characteristic and (**b**) precision–recall curves for logistic regression, random forest, and gradient-boosted trees under protein-group-aware cross-validation. Labels derive from curated actin-regulator annotations; features are independent of these labels. Logistic regression performed best (ROC-AUC 0.66; average precision 0.24 vs. a 0.05 baseline). Modest performance reflects the imbalanced task and indicates the analysis is suited to prioritization rather than definitive classification.

Although IP-MS identified 47 high-confidence US3-associated proteins, not all interactors are expected to contribute equally to F-actin remodeling, and manual ranking of dozens of candidates is subjective. The classifiers were trained and evaluated using the broader 639-protein dataset, while the 47 proteins meeting the high-confidence IP-MS criteria constituted the principal experimentally supported US3-interactor subset. Predictions involving proteins outside this subset were interpreted as computational prioritizations rather than evidence of physical association with US3. We therefore sought an objective, reproducible method to prioritize interactors whose quantitative proteomic signatures most closely resemble established actin regulators, in order to nominate the most promising candidates for downstream biological validation. The classifier was not intended to independently discover novel biology, but to prioritize experimentally detected interactors for subsequent functional investigation. We framed this as supervised classification and trained three classifiers—logistic regression (LR), random forest (RF), and gradient-boosted trees (GB) using only label-independent quantitative mass-spectrometry features, with curated actin regulator membership as the supervised target. Annotation-derived variables were deliberately excluded so that predictions depended solely on experimental evidence and to avoid the circularity between features and labels noted during review. Representative ROC and precision–recall curves are shown in Figure 5. Per-fold metrics for all three classifiers are provided in Supplementary Data.

**Figure 6:**
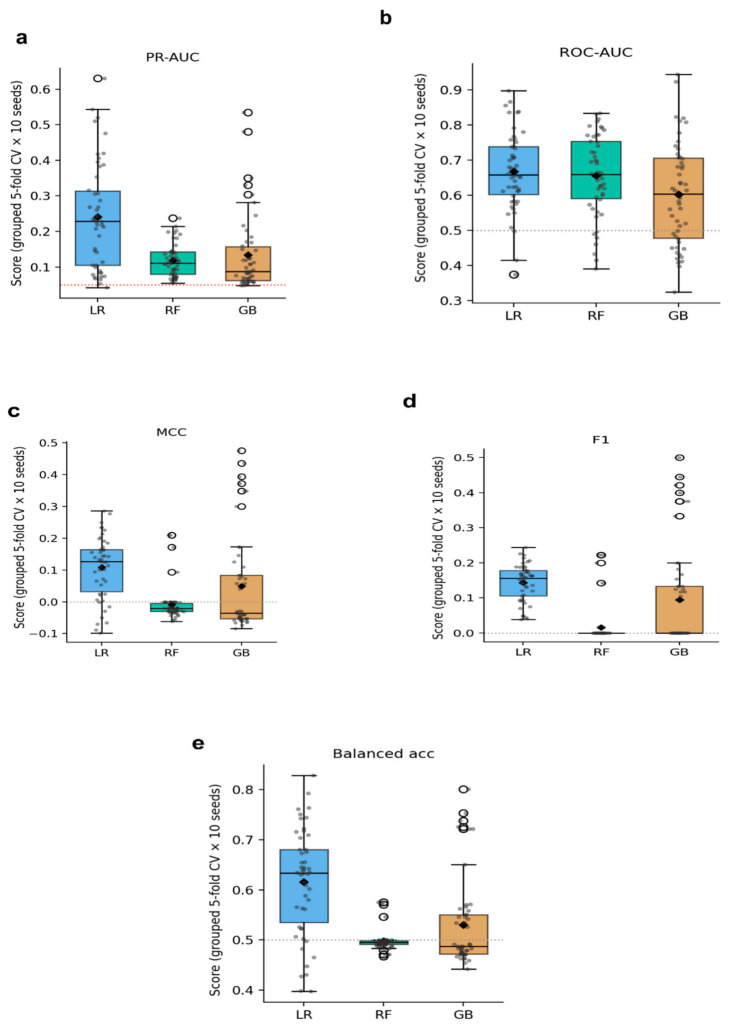
Model comparison under protein-group-aware cross-validation. Box-and-whisker plots summarizing performance across 10 random seeds for logistic regression (LR), random forest (RF), and gradient-boosted trees (GB). (**a**) Average precision (AP). (**b**) Area under the ROC curve (ROC-AUC). (**c**) Matthews correlation coefficient (MCC). (**d**) F1 score. (**e**) Balanced accuracy. Logistic regression significantly outperformed both tree-based models on average precision (Friedman χ^2^ = 29.6, *p* < 0.0001; Wilcoxon *p* < 0.0001 for LR vs. RF and LR vs. GB), whereas random forest and gradient boosting did not differ (*p* = 0.65). The dashed line indicates the class-prevalence baseline (AP = 0.05).

Under protein group aware cross validation, all three classifiers exceeded the class-prevalence baseline (average precision 0.05), and logistic regression performed best and most stably (AP 0.24; ROC-AUC 0.66), significantly outperforming random forest and gradient boosting (Friedman χ^2^ = 29.6, *p* < 0.0001; Wilcoxon *p* < 0.0001 for both pairwise comparisons; random forest vs. gradient boosting not significant, *p* = 0.65). Tree-based models fell to near-baseline average precision (AP ≈ 0.09–0.12) once identical-profile proteins were constrained to the same fold, indicating that their apparent advantage under ordinary cross-validation reflected duplicated mass-spectrometry profiles rather than generalizable signal. Because only ~5% of proteins belonged to the positive class, a random classifier would achieve an average precision of ~0.05; the observed AP of 0.24 therefore represents roughly a five-fold enrichment over random ranking. The pooled out-of-fold precision–recall curve yielded an AP of 0.17, whereas the mean AP across grouped cross-validation folds and random seeds was 0.24. The superior performance of logistic regression over the tree-based models is consistent with a largely simple, approximately linear separation between regulators and non-regulators in the quantitative proteomic feature space, and with the limited number of positive examples, which can disadvantage more flexible models. Notably, even using only quantitative proteomic measurements, the ranking was enriched for proteins involved in Rho-family signaling and actin nucleation and remodeling, indicating that these experimental features carry real, if partial, information about actin regulatory identity, sufficient to prioritize detected interactors for validation rather than to classify them definitively, and cross-validated performance distributions across models are summarized in Figure 6. Full per-fold and multi-seed metrics are provided in Supplementary Data.

**Figure 7:**
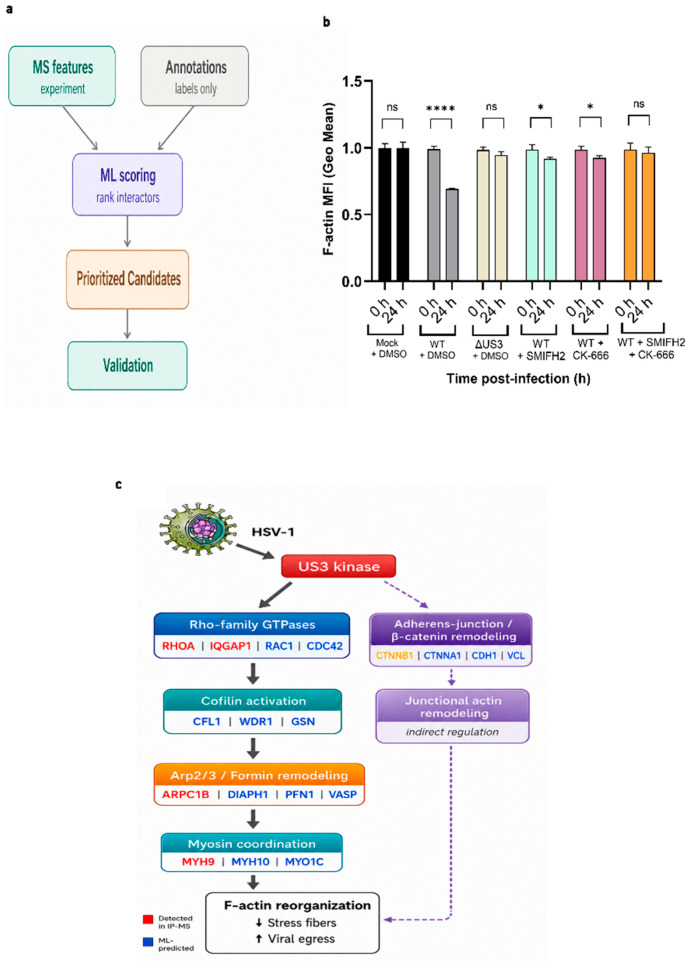
Pharmacological validation and inferred model of US3-mediated actin remodeling. (**a**) Schematic of the machine-learning workflow used to prioritize candidate F-actin regulators. Label-independent quantitative mass-spectrometry features were used for model training, whereas curated actin-regulator annotations were used only to define class labels. Prioritized candidates were subsequently selected for biological interpretation and experimental validation. (**b**) Flow-cytometric quantification of F-actin levels in N2A cells infected with WT HSV-1 or ΔUS3 HSV-1 and treated with the formin inhibitor SMIFH2, the Arp2/3 inhibitor CK-666, or both inhibitors. F-actin abundance was measured as the GeoMean MFI of Alexa Fluor 647–phalloidin at 0 and 24 hpi. Inhibition of either pathway partially attenuated the WT-associated reduction in F-actin, whereas combined inhibition prevented a statistically detectable reduction under the tested conditions. Data represent the mean ± SEM from three independent biological experiments. Statistical significance was assessed using two-way ANOVA followed by Šidák’s multiple-comparisons test. ns, not significant; * *p* < 0.05; **** *p* < 0.0001. (**c**) Proposed model integrating the US3 IP-MS interactome, machine-learning-prioritized proteins, and experimentally observed F-actin dynamics. Proteins detected in the US3 IP-MS interactome are distinguished from computationally prioritized or inferred proteins. Arrows represent proposed regulatory relationships, many of which may be indirect. The association between US3 and β-catenin was independently validated by reciprocal co-immunoprecipitation (Figure 4). During early infection, US3 is proposed to promote actin-polymerization programs involving Arp2/3- and formin-associated pathways, potentially supporting intracellular transport. At later stages, US3 is proposed to modulate Rho-family GTPase and cofilin signaling, contributing to stress-fiber disassembly and cytoskeletal remodeling associated with viral egress and spread. Myosin-associated motor complexes may contribute to cytoskeletal organization during both phases.

Prioritized proteins included both interactors detected in the IP-MS dataset (e.g., RHOA, MYH9) and proteins present only as computationally predicted regulators that were not co-purified (e.g., RAC1, CDC42, CFL1, PFN1, VASP, DIAPH1); we distinguish these two categories explicitly to avoid conflating experimentally detected interactors with predicted regulators. These predictions align with our experimental observation that WT HSV-1 induces pronounced F-actin remodeling at 24 hpi while ΔUS3 fails to disrupt stress fibers. One interactome-prioritized candidate, β-catenin (CTNNB1), was independently corroborated by reciprocal co-immunoprecipitation (Figure 4c), supporting a working model in which US3 engages Rho-family signaling and cofilin-mediated severing while simultaneously engaging Arp2/3 and formin nucleation machinery to coordinate spatial remodeling (Figure 7a). Validation of the remaining prioritized candidates is the subject of ongoing work.

### 3.5. Pharmacological Inhibition of Arp2/3 and Formin Pathways Attenuates US3-Dependent F-Actin Remodeling

Together, these results indicate that quantitative proteomic features alone contain sufficient information to enrich for candidate actin regulators, enabling systematic prioritization of US3-associated proteins for downstream validation. Because this prioritization highlighted proteins associated with Arp2/3- and formin-dependent actin regulation, we next tested whether these pathways contribute functionally to US3-mediated F-actin remodeling, inhibiting formin-mediated filament elongation and Arp2/3-mediated nucleation with selective small-molecule inhibitors. N2A cells were infected with WT HSV-1 or deltaUS3 and treated with the formin inhibitor SMIFH2 (20 μM), the Arp2/3 inhibitor CK-666 (100 μM), or both, at concentrations previously shown to inhibit formin- and Arp2/3-dependent actin polymerization without significant cytotoxicity [35,36,37]. Under these inhibitor conditions, ΔUS3 HSV-1 produced single-cycle titers comparable to wild-type virus at 12 and 24 hpi (Supplementary Figure S1; MOI 5, plaque assay; two-way ANOVA with Šidák’s multiple comparisons test, *p* > 0.05), indicating that US3 is dispensable for bulk single-round replication and that its actin-regulatory activity instead operates at the level of cytoskeletal remodeling relevant to viral egress and cell-to-cell spread. DMSO-treated mock cells showed no detectable change in F-actin over the 24 h experimental window (deltaGeoMean = 0.000; *p* > 0.9999). F-actin levels were quantified by flow cytometry using Alexa Fluor 647-phalloidin and reported as GeoMean MFI at 0 and 24 hpi (Figure 7b).

WT HSV-1 infection produced a robust reduction in F-actin by 24 hpi (deltaGeoMean = 0.2973; 95% CI 0.2445–0.3502; **** *p* < 0.0001), whereas deltaUS3-infected cells showed no significant change (deltaGeoMean = 0.0377; 95% CI −0.0152 to 0.0905; *p* = 0.1544), confirming that US3 expression is required for late-stage actin disassembly. Treatment of WT-infected cells with SMIFH2 significantly reduced the magnitude of F-actin depletion at 24 hpi (deltaGeoMean = 0.0690; 95% CI 0.0161–0.1219; * *p* = 0.0127), and CK-666 produced a comparable attenuation (deltaGeoMean = 0.0597; 95% CI 0.0068–0.1125; * *p* = 0.0286). Simultaneous inhibition of both pathways completely abolished the US3-dependent reduction in F-actin (deltaGeoMean = 0.0247; 95% CI −0.0282 to 0.0775; *p* = 0.3452). These findings provide experimental support for the regulatory network identified by the interactome and classifier analyses, demonstrating that HSV-1 US3 engages multiple actin regulatory complexes to coordinate late-stage F-actin disassembly during infection.

## 4. Discussion

This study provides a systems-level view of how the HSV-1 US3 kinase engages conserved host actin regulatory complexes to drive biphasic F-actin remodeling during infection. By integrating quantitative flow cytometry, high-resolution confocal imaging, global interactomics, protein complex annotation, machine-learning classification, and pharmacological perturbation, we identify a coordinated network of US3-associated actin regulatory factors required for the cytoskeletal reorganization that accompanies HSV-1 egress and spread. While individual US3 mediated effects on cofilin and PAKs have been described in PRV and HSV systems, the present work extends those findings in three important ways: by quantitatively defining the temporal pattern of US3-dependent F-actin dynamics in infected cells, by demonstrating that US3 interfaces with conserved multiprotein machines rather than with isolated host factors, and by experimentally validating the requirement for Arp2/3- and formin mediated polymerization activities in US3-driven F-actin remodeling.

Flow cytometry established that US3 drives dynamic, biphasic regulation of F-actin during infection (Figure 1). WT HSV-1 increased F-actin at 6 hpi, consistent with a polymerization phase that supports viral transport, and subsequently reduced F-actin by 12–24 hpi. deltaUS3-infected cells remained indistinguishable from mock at all time points, demonstrating that US3 is required for both the early accumulation and the late disassembly phases. Confocal microscopy supported these findings (Figure 2a–f). Quantitative image analysis reinforced the flow-cytometry results: WT HSV-1 drove a pronounced, US3-dependent reduction in both F-actin content and area at 24 hpi, with ΔUS3 largely phenocopying mock. The residual, US3-independent decrease in area fraction observed in ΔUS3-infected cells suggests that infection contributes a minor cytoskeletal effect through US3-independent pathways, while the dominant remodeling of F-actin content requires US3 (Figure 2g,h).

This temporal pattern is mechanistically consistent with the published literature on alphaherpesviruses. In pseudorabies virus (PRV), US3 was shown to activate the PAK–LIMK pathway, leading to cofilin phosphorylation and breakdown of actin stress fibers [16,17,38,39]. Similarly, HSV-1 US3 has been shown to induce actin rearrangements and membrane protrusions that facilitate cell-to-cell spread [14,19,40]. However, these prior studies did not resolve whether US3 promotes early actin stabilization prior to late disassembly. Our data demonstrate that US3 does not simply depolymerize actin but instead orchestrates a temporal switch in its regulatory state: early accumulation supports capsid transport, organelle repositioning, and assembly of cortical platforms for virion trafficking, while late disassembly facilitates nuclear lamina deformation (in coordination with US3-mediated lamin phosphorylation), reduction in cortical stiffness for secondary envelopment and egress, and formation of membrane protrusions for spread.

Proteomic analysis identified 47 high-confidence US3 interactors, and ClueGO and g:Profiler enrichment analyses revealed dominant clustering in actin nucleation, polymerization, severing, and cortical cytoskeleton organization pathways (Figure 3). Mapping US3 interactors onto the CORUM database showed that these regulators are embedded in conserved multiprotein assemblies rather than acting as isolated components (Figure 4). US3 engaged multiple subunits of Arp2/3 nucleation, formin-containing elongation, and cofilin-associated severing complexes, as well as vesicle and organelle transport machineries including kinesin-1, dynein, COPI, V-ATPase V1, nuclear pore, and TIM/TOM complexes. This complex-level engagement allows a single viral kinase to coordinately tune nucleation, elongation, and severing of F-actin while controlling long-range vesicular transport and organelle positioning, providing signal amplification (phosphorylation of one subunit may reprogram an entire complex), spatial precision (complexes localize to defined cellular compartments), and functional integration of actin remodeling with vesicle trafficking. These principles parallel strategies observed in other viruses, including HIV-1 recruitment of ESCRT machinery via Gag and HSV-1 engagement of ESCRT-III and VPS4 for cytoplasmic envelopment, but have not previously been demonstrated for HSV-1 US3 at this systems level [41,42,43,44]. The physical association of US3 with a prioritized interactor was confirmed biochemically by reciprocal co-immunoprecipitation of US3 and β-catenin (CTNNB1) (Figure 4c). This validation, at the adherens–junction interface, supports the biological relevance of the US3 interactome and extends the model to junction-associated actin remodeling relevant to cell-to-cell spread.

Integrating the interactome, complex mapping, and machine-learning analyses, we propose a hierarchical model in which US3 modulates Rho-family GTPase signaling to activate cofilin-mediated actin severing while simultaneously engaging Arp2/3 nucleation, formin elongation, and motor complexes to coordinate spatial remodeling (Figure 7a). Cofilin is a well-established actin-severing factor whose activity is controlled by phosphorylation at Ser3 via LIM kinase downstream of RhoA/PAK signaling [15,17,18]. Published PRV studies demonstrated US3-dependent modulation of cofilin phosphorylation, but whether HSV-1 US3 engages upstream Rho GTPases during authentic infection was previously unclear. Our integrated data are consistent with an inferred regulatory axis in which US3 modulates Rho GTPase signaling and the cofilin activation state to promote F-actin severing. Of these nodes, RHOA and MYH9 were detected as IP-MS interactors, whereas RAC1, CDC42, and CFL1 were computationally predicted regulators that were not co-purified; the axis therefore integrates interactome, prediction, and prior literature rather than representing a directly demonstrated cascade. Under protein-group-aware cross-validation with label-independent mass-spectrometry features, logistic regression provided the best prioritization (average precision 0.24; ROC-AUC 0.66), significantly exceeding random forest and gradient boosting (Figure 5 and Figure 6); because the positive class is small (~5%), performance is reported as average precision and MCC rather than accuracy. The modest performance indicates the machine learning is best interpreted as interactor prioritization, consistent with the independent biochemical validation of a prioritized interactor, β-catenin (CTNNB1) [44,45].

Early in infection, US3 likely activates PAK–LIMK signaling, leading to cofilin phosphorylation and inactivation, while simultaneously engaging Arp2/3, formins, VASP, and profilin to promote actin nucleation and elongation. This would favor the assembly of cortical and perinuclear F-actin, consistent with the initial F-actin increase observed by flow cytometry and the organized stress fibers seen in early confocal images (Figure 1 and Figure 2). Such cortical platforms have been implicated in capsid trafficking and organelle positioning, both of which support productive HSV-1 infection. In parallel, HSV-1 nuclear replication compartments exhibit condensate-like properties, with ICP4 and other viral replication proteins contributing to a dynamic organization consistent with liquid–liquid phase separation [46]. US3 directly phosphorylates lamin A/C, destabilizing the nuclear lamina to facilitate capsid transit through weakened perinuclear barriers, a process that is mechanistically amplified by late-stage F-actin disassembly, which reduces cytoskeletal tension on the nuclear envelope [47,48,49]. Concurrent engagement of motor and vesicular transport complexes positions US3 to synchronize actin severing with microtubule-based virion trafficking and vesicle maturation, ensuring envelopment at actin-depleted membrane sites permissive for exocytosis [35,36,37,50]. At later stages, US3 reconfigures this network by modulating Rho-family GTPase signaling and switching cofilin and other severing factors such as gelsolin and WDR1 into their active states. The resulting severing and depolymerization of cortical actin opens physical routes at the nuclear envelope and plasma membrane, enabling capsid egress through US3-phosphorylated lamina gaps and facilitating vesicle fusion at sites of reduced cortical stiffness. In this view, US3 functions as a kinetic regulator of cytoskeletal plasticity rather than as a unidirectional depolymerizing factor, a property particularly well suited to a viral life cycle in which cytoskeletal architecture must be reorganized multiple times in a temporally ordered fashion.

Pharmacological inhibition of formin and Arp2/3 pathways confirmed the functional relevance of these complexes (Figure 7b). Combined SMIFH2 and CK-666 treatment abolished US3-dependent F-actin reduction, while individual inhibitors produced partial attenuation, indicating that both nucleation systems contribute to the US3-driven phenotype and that their activities are required in parallel rather than redundantly. The additivity of the two inhibitors is consistent with the CORUM analysis showing that US3 engages Arp2/3 and formin-associated complexes at distinct sites, and with the broader interactome architecture in which US3 simultaneously interfaces with multiple polymerization, severing, and motor systems. Notably, the deltaUS3 mutant phenocopied combined pharmacological inhibition, recapitulating the absence of late-stage F-actin remodeling, which reinforces the conclusion that US3 functions upstream of both nucleation systems rather than within a single linear pathway. The convergence of interactome, classifier, and pharmacological data on a small set of actin regulatory complexes provides a coherent framework for understanding how HSV-1 US3 hijacks conserved cytoskeletal machinery during infection, and identifies a tractable set of host targets—Arp2/3, formin-associated, cofilin-related, and Rho-family-associated complexes—whose contributions to HSV-1 egress and spread can now be dissected at the level of individual subunits.

Several limitations are identified, suggesting future investigations. Direct measurement of Rho-family GTPase activation (e.g., by G-LISA or pull-down assays) and cofilin Ser3 phosphorylation during WT versus deltaUS3 infection, together with phosphoproteomic comparison of WT and kinase-dead US3, will be needed to formally establish the proposed signaling cascade and to distinguish kinase-dependent from scaffolding contributions of US3. Although Vero cells were chosen for IP-MS because of their high HSV-1 yield and N2A cells for imaging as a neuronal model relevant to HSV-1 biology, confirmation of the interactome in a single human-derived system would strengthen the work; peptide identification against the human reference proteome may also under-detect Vero-specific variants. The machine-learning analysis used a relatively small positive class defined using annotation-derived labels. Although annotation-derived variables were excluded from the model features, class imbalance, potential label uncertainty, and the limited number of positive examples constrain model generalizability. Therefore, the model performance is best interpreted as prioritization for experimental follow-up rather than as evidence of de novo discovery. SMIFH2 and CK-666 can have off-target effects, and genetic perturbation of individual subunits of Arp2/3, formins, and cofilin will help resolve the contribution of each complex. Finally, our findings are based on a single neuronal cell line and a Vero-based interactome at one infection timepoint; time-resolved interactomics and analyses in epithelial and immune cell types will be important to define how the US3–host complex engagement evolves across the infection cycle and across the cell types encountered in vivo.

Despite these caveats, our data collectively establish US3 as a central organizer of host actin regulatory complexes rather than a kinase acting on a few isolated substrates. By targeting intact host complexes, US3 achieves coordinated remodeling of actin architecture, revealing an evolutionarily refined strategy of cytoskeletal hijacking that integrates kinase activity with the conserved machinery governing actin nucleation, severing, and vesicular trafficking. The breadth of complexes engaged, spanning cytoskeletal nucleation, motor-driven transport, vesicular acidification, and nuclear pore function, suggests that US3 acts as a coordinating hub that synchronizes diverse host processes required for productive HSV-1 infection. These insights provide a conceptual foundation for therapeutic targeting of host regulatory hubs to limit HSV-1 dissemination, and they nominate Arp2/3, formin, cofilin, and Rho-family-associated complexes as priority cellular targets for antiviral strategies aimed at the late-stage viral egress and cell-to-cell spread that drive HSV-1 pathogenesis.

## Data Availability

Processed interactome datasets and machine-learning performance results are provided in the Supplementary File. Supplementary Data S4 contains the feature matrix and class labels used for machine-learning classification and training. Additional data supporting the findings of this study are available from the corresponding author upon reasonable request.

## Supplementary Materials

The following supporting information can be downloaded at: https://www.mdpi.com/article/10.3390/v18070793/s1.
