# Supplementary material for: HSV-1 US3 Hijacks Conserved Actin Regulatory Complexes to Drive F-Actin Remodeling
Source: Viruses. 2026 Jul 19;18(7):793. doi: 10.3390/v18070793 (PMC13431605; doi:10.3390/v18070793)
Supplement: Supplementary file 1 [file viruses-18-00793-s001.zip › Supplementary File.pdf]

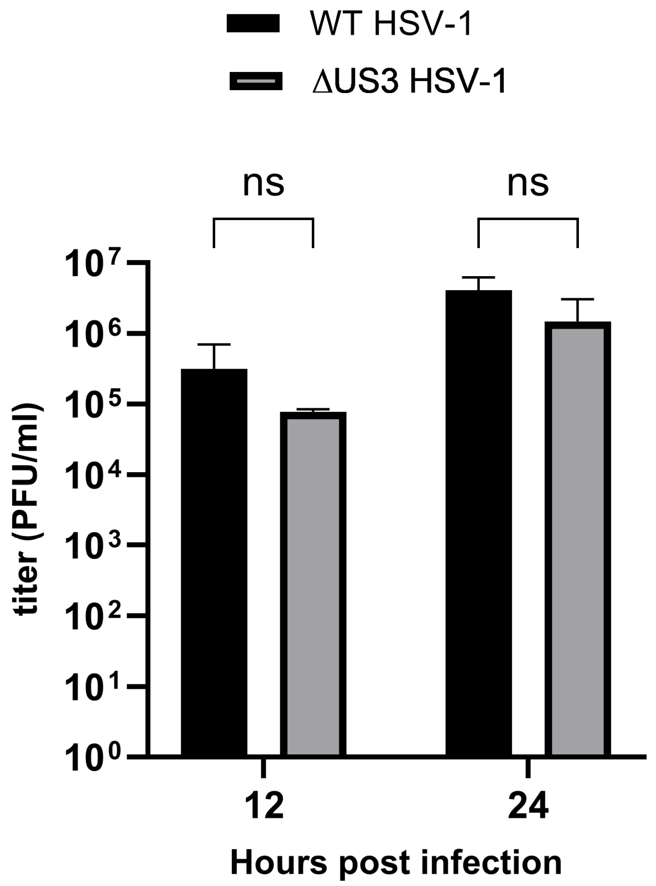

Figure S1. Wild-type and ΔUS3 HSV-1 display comparable single-cycle replication kinetics in N2A cells under actin inhibitor treatment. N2A (mouse neuroblastoma) cells were pretreated with the Arp2/3 complex inhibitor CK-666 (100 μM) and the formin inhibitor SMIFH2 (20 μM) for 30 minutes prior to infection with wild-type HSV-1 (McKrae strain) or the ΔUS3 mutant at a multiplicity of infection (MOI) of 5 to ensure synchronous, single-cycle infection. Inhibitors were maintained in the culture medium throughout the infection period. At 12 and 24 hours post infection (hpi), cells and supernatants were harvested, and infectious virus was quantified by plaque assay on Vero cells. Data are presented as mean ± SD of three independent biological replicates. Statistical significance was evaluated by two-way ANOVA with Šídák's multiple comparisons test (ns, not significant; $p > 0.05$). No significant difference in viral titer was detected between WT and ΔUS3 HSV-1 at either timepoint (12 hpi, $p = 0.9721$; 24 hpi, $p = 0.0821$), indicating that deletion of US3 does not impair productive viral replication in N2A cells under these conditions, and that combined CK-666 and SMIFH2 treatment does not exert non-specific antiviral effects on HSV-1 replication.

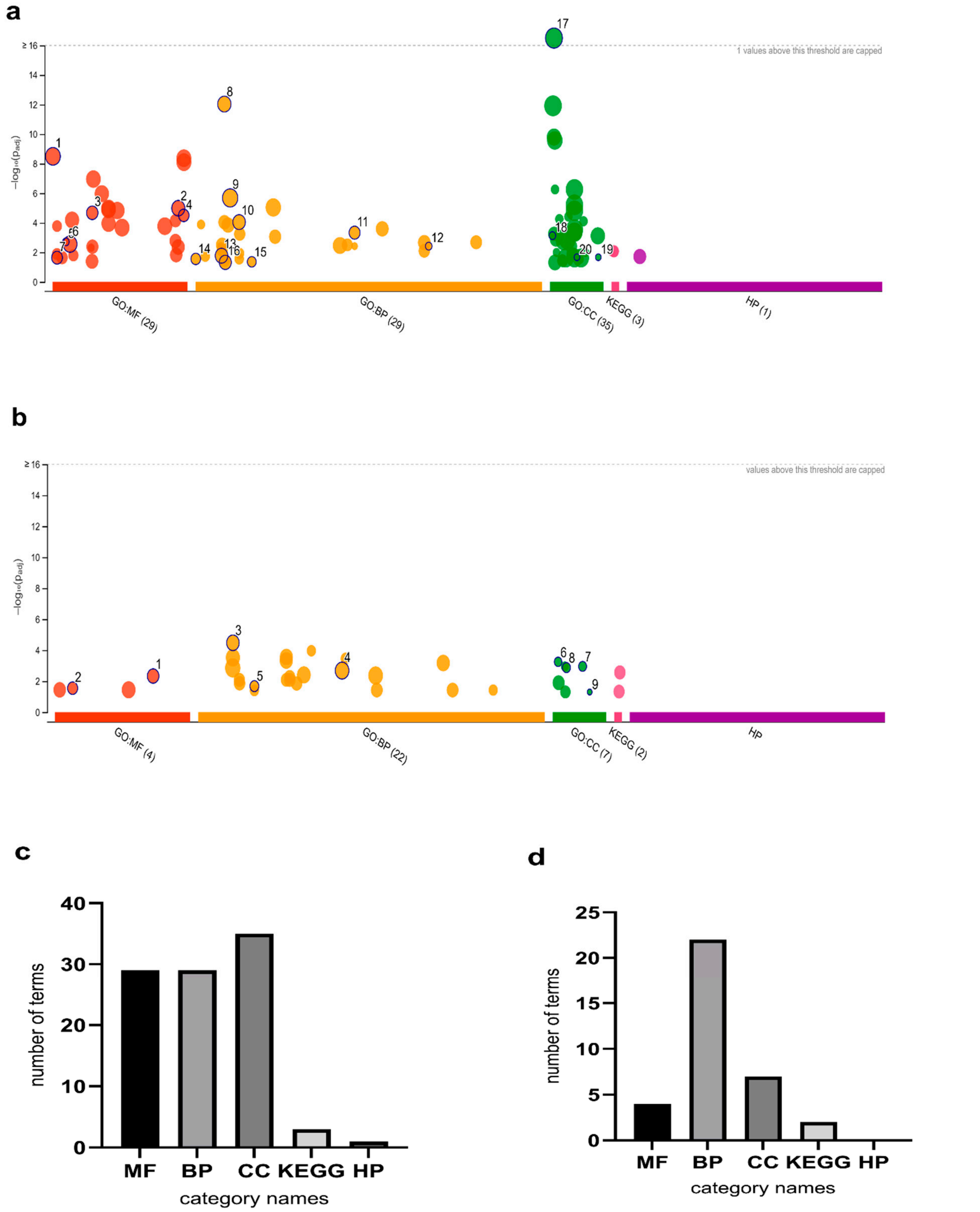

Figure S2. g:Profiler enrichment shows that US3 interactors are strongly engaged with actin related functions. (a) Enrichment plot for all US3 interactors. Dots represent significantly enriched GO/KEGG/HP terms positioned by functional category (MF, BP, CC,

KEGG, HP) on the x-axis and $-\log_{10}$(adjusted p-value) on the y-axis. (b) Enrichment plot restricted to actin-related US3 interactors. Actin cytoskeleton terms become further concentrated and more significant. (c) Bar graph summarizing the number of significant terms per category for panel (a) (all interactors). (d) Bar graph summarizing the number of significant terms per category for panel (b) (actin-related subset).

a b

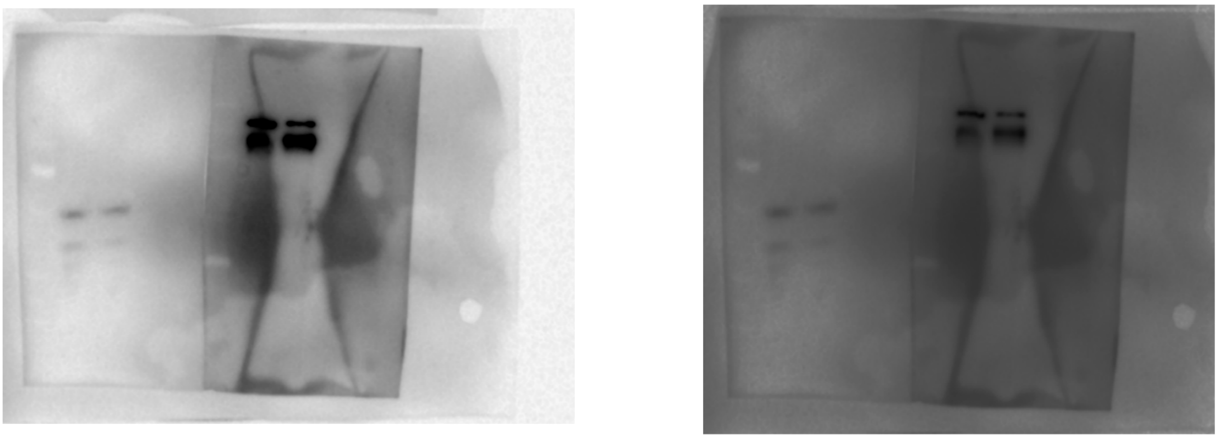

Figure S3. Reciprocal co-immunoprecipitation of HSV-1 US3 and β-catenin. The uncropped Western blot is shown with uniform automatic brightness adjustment (a) and without brightness adjustment (b).

Supplementary Tables

Table S1: Oligonucleotides used in this study to create mutant virus

Oligonucleotide name	Purpose	Length (bp)	Sequence (5′–3′)
US3-up-Fnull	Upstream homology arm for markerless deletion of the US3 open reading frame	79	AAAACCCCGGGGCCCGTCGTTCGGGGTGCTCGTTGGTTGGCACTCACGGTGCGGCGAAGGATGACGACGATAAGTAGGG
US3-dwn-Rnull	Downstream homology arm for markerless deletion of the US3 open reading frame	127	GTCGGGGTCTTTTTGTGCCAACCCGCAAACAGCACCGCCCCCGGGGGCGGTCGCCGCACCGTGAGTGCCAACCAACGAGCACCCGAACGACGGGCCCCGGCAACCAATTAACCAATTCTGATTAG

Table S2: Antibodies and reagents

Name	catalog
Rabbit anti-US3	Abcam, ab92392
β-catenin antibody	9562
SMIFH2	Medchemexpress: HY-16931
CK-666	Medchemexpress: HY-16926
Viobility™ 405/520 Fixable Dye	Miltenyi Biotec; Cat: 130-109-814
Alexa Fluor 647–conjugated phalloidin	Thermo Fisher Scientific, A22287
Protein G magnetic Dynabeads	10007D

Supplementary Data Descriptions

1. File Name: Supplementary Data S1

Description: 2way ANOVA of F actin Geomean score

2. File Name: Supplementary Data S2

Description: 2way ANOVA of F actin Area Fraction

3. File Name: Supplementary Data S3

Description: 2way ANOVA of MFI

4. File Name: Supplementary Data S4

Description: ML Feature Matrix

5. File Name: Supplementary Data S5

Description: ML grouped allseeds metrics

6. File Name: Supplementary Data S6

Description: ML grouped per fold metrics
